# Research on Microscopic Properties of TiBw/TC4 Composites for Drilling Process

**DOI:** 10.3390/ma12132112

**Published:** 2019-06-30

**Authors:** Yong Feng, Binghui Jia, Xiaoyu Wang, Min Zhang, Zihao Zhu

**Affiliations:** Department of Mechanical Engineering, Nanjing Institute of Technology, Nanjing 211100, China

**Keywords:** TiB_W_/TC4 composites, drilling, microscopic properties, failure, whisker rotation

## Abstract

TiB-whisker-reinforced TiBw/TC4 composites are widely used in aviation, aerospace, automotive, and various other industries. However, the drilling force and temperature have a large effect on the drilling micromechanical properties of TiBw/TC4 composites. In order to explore the micro-mechanical properties and promote the optimization of drilling process, the representative volume elements of TiB_W_/TC4 composites were established in the Digimat-FE software tool to determine the micromechanical properties and failure mechanism of TiBW/TC4 composites. The results show: (1) maximum stress occurs at the whiskers, whereas the minimum stress appears within the basket matrix region of the reinforced phase. (2) When the force reached 300 N and the temperature reached 75 °C, the junction between the whiskers and the matrix firstly failed; when the force reached 525 N and the temperature reached 112 °C, the matrix began to fail; when the force reached 675 N and the temperature reached 138 °C, the whiskers failed. (3) The main reason for the failure of the junction between the matrix and the whiskers was that the shear stress exceeded the connection strength and the axial stress accelerated the failure process. Displacement and rotation of the whiskers were shown to occur at the moment of junction failure, owing to differences in the stress experienced on different sides of the material.

## 1. Introduction

Compared with other matrix titanium alloys, titanium matrix composites (TMCs) have high specific stiffness, high specific strength, excellent high temperature performance and wear resistance. The research and application of composite materials have become a hot spot for high-tech advantages and as an advanced composite material will gradually replace some of the traditional metal materials used in the aviation, aerospace, and automobile industries. However, the significant enhancement of TMCs and the unique problems of the cold-work hardening and difficult to dissipate heat in the drilling process multiply the difficulty of machining.

In recent years, research on the properties of TMCs has mainly been carried out in relation to two aspects -mechanical analysis of the microstructure and optimal simulation of the machining process [1,2,3,4,5]. In the aspect of microstructure analysis, the research mainly focuses on the reinforced phase of composites. Kuang et al. [6] researched the strengthening effect of TiB whiskers on titanium matrix composites. The results showed that the strength and room temperature tensile strength of composites increase with the increase of the reinforced phase. The strengthening effect of TiB whiskers was mainly reflected in three aspects, namely bearing stress, refining the matrix structure and reducing the oxygen content in the matrix alloy. Zhao et al. [7] researched the surface topography of titanium matrix composites during grinding. They found that the surface quality of composites with the reinforced phase grain size of 40 μm was significantly higher than that of 80 μm. This had a great influence on the increase of mechanical properties of composite materials and the quality of process. Hong et al. [8] analyzed the plasticity effect of particle-reinforced titanium matrix composites. When the size ratio of the matrix powder to the reinforcing phase particles was 40:1, the composite material was basically non-plastic. When the size ratio dropped to 3:1, the elongation of the material was increased by nearly 10 times, which was very beneficial to machining. Moreover, simulation analysis is mainly conducted to find and improve the processing defects of materials. Wang et al. [9] established a finite element model of a SiC particle reinforced aluminum matrix composite with high volume fraction and random distribution, and applied a milling simulation to it. The results showed that the rotation and pulling of SiC particles were the main causes of defects such as cracks and delamination of material. The conclusion was verified by comparison with the surface profile after the experiment. Fernandes et al. [10] built a three-dimensional dynamic model of the composite to simulate the drilling process. The results showed that the composites were less stressed and strained with the higher feed rate. Wang et al. [11] developed a multi-phase two-dimensional finite element model of aluminum-based composites, which was applied to high-speed milling simulation. It revealed the milling removal mechanism of aluminum matrix composites through the matrix, enhanced phase, miscellaneous interactions and complex stress distribution caused by the cutting area. Isbilir et al. [12] established a three-dimensional finite element model for drilling carbon fiber-reinforced composites according to the given bit geometry and process parameters, with considering a three-dimensional progressive intra-layer failure model based on Hashin theory. The results showed that the model could predict the induced thrust, torque, damage area and stratified areas generated by carbon fiber reinforced polymer drilling. Xin et al. [13] derived the analytical solution of the plastic zone at the crack tip of the composite based on the Tsai–Wu criterion. It showed that the shape of the plastic zone at the tip of the crack was closely related to the mechanical properties of the material and the depth of the crack was related to the applied drilling stress.

In summary, the shape size, volume fraction of a particular enhancement phase, and reasonable cutting force and cutting temperature are all beneficial to improve the processing quality of materials. Drilling research on titanium matrix composites using simulation software can provide guidance for drilling experiments. However, the literature on the micromechanical study of titanium matrix composites with reinforced phases is rarely reported, which has important practical significance for how to reduce the difficulty and mechanical efficiency of mechanical processing of reinforced phase composites. Therefore, TiBw/TC4 composites with 8.5% volume fraction were used as research objects and Digimat multi-scale modeling technology was used to establish multi-scale representative voxels that can accurately reflect the structural characteristics of materials. By simulating the drilling load and analyzing the effects of drilling force and temperature change on the stress and strain of the composite, the structural failure process and failure mode of the composite can be explored during the drilling process. The finite element simulation and the results of the drilling experiment were compared and verified. Theory and data support for optimizing the processing technology of TiBw/TC4 composite materials during drilling are provided by solving the difficult processability of titanium matrix composites from the microcosmic property of the materials themselves.

## 2. Finite Element Analysis of TiBw/TC4 Composites

### 2.1. Research Scheme

**Figure 1:**
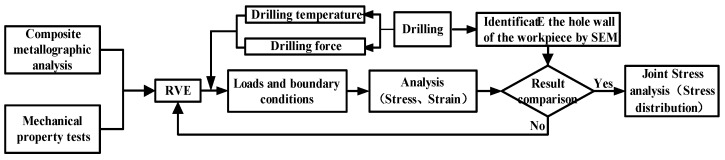
Flow chart of the method used to analyze microscopic thermodynamic characteristics.

The FE module in Digimat software (e-Xstream, MSC, Antwerp, Belgium) was selected. Its built-in solver of the module is a solver based on Marc software, which adopts a CASI iterative algorithm. The simulation time was set to 1 s (the stage when the drilling force increasing rapidly to its maximum) with a time increment of 0.003 s, and the solution precision was set to 10^−8^. For better simulation of drilling conditions, 60% of the drilling force was taken as the axial drilling force, and the remaining 40% was applied to the model as the shearing drilling force according to the Zitoun’s drilling force model [14]. The thermal stress loading of the model was selected by Biot’s modified Fourier heat conduction equation, and thermal stress in all directions can be calculated by using a stress-strain constitutive equation according to the obtained displacement according to the thermal expansion coefficient of the model. TiBw/TC4 composites belong to short-fiber reinforced materials with the isotropy, therefore, the Tsai-Wu failure criterion was selected to evaluate the combined influence of all stress components on material failure. Moreover, it also can appropriately describe the comprehensive plastic failure properties of composites. The stress and strain distribution of TiBw/TC4 composites under different drilling forces and temperature loads can be obtained through the above methods, including equivalent stress, shear stress, axial stress, and the strain of the phase and matrix. According to the variation law of stress and strain, the structural failure process of composite materials under drilling conditions can be explored. The analysis process is presented in Figure 1.

### 2.2. Modeling Parameters of the Model

#### 2.2.1. Micro Structure of TiBw/TC4 Composites

**Figure 2:**
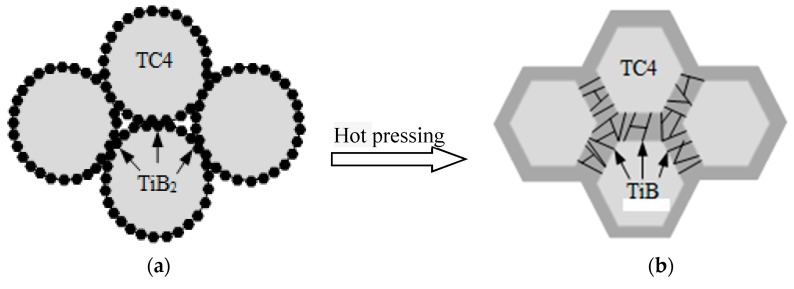

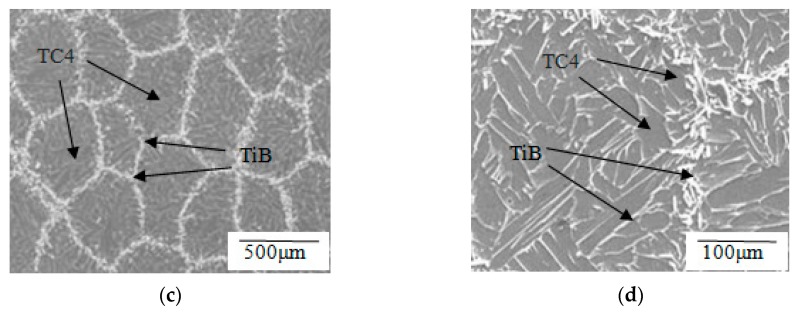
Composite reaction diagram and scan photos for 8.5 vol% TiBw/TC4. (**a**) Before hot pressing reaction; (**b**) After hot pressing reaction; (**c**) Low magnification scan photo; (**d**) High magnification scan photo.

Based on the volume fraction 8.5% TiB whisker-reinforced titanium matrix composites in the literature [15], spherical TC4 powder with a particle size of 120–220 μm and TiB_2_ powders with a particle size of 1–5 μm were mixed at a certain ratio, then TiB_w_/TC4 composites were prepared by high temperature hot pressing sintering. The TC4 powder softened and densified between the particles due to the high temperature and the original spherical TC4 powder became a polyhedron. TiB_2_ reacted with Ti to form whisker-like TiB. Due to the effect of thermal diffusion, some of the TiB whiskers grow into the interior of the matrix like nail tips, which play an important role in the connection of the matrix at both ends. The reaction process diagram is presented in Figure 2a,b. The microstructure of the material was observed by scanning electron microscope (SEM, JEOL JSM-6300LV, Tokyo, Japan), as shown in Figure 2c,d. In the figure, the TC4 matrices are distributed in a hexahedron shape, and the TiB whiskers are distributed in a continuous network. The phase identification of the composite material showed that TiB_2_ was not found, indicating that the TiB2 powder completely reacted with Ti in TC4 to form TiB whiskers.

#### 2.2.2. Performance and Damage Evolution Parameters of TiBw/TC4 Composites

The performance parameters of the TC4 matrix, TiB whiskers and the damage evolution parameters of Tsai-Wu failure criterion are shown in Table 1 [16,17,18].

#### 2.2.3. Simulation Loads of TiBw/TC4 Composites

**Figure 3:**
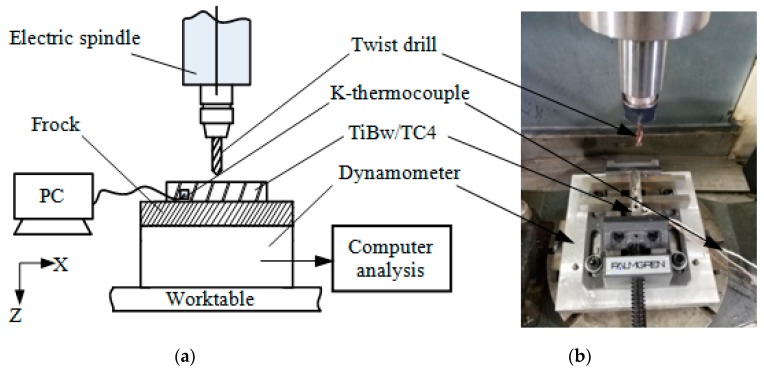
Drilling experiments on TiBw/TC4 composite. (**a**) Schematic diagram of drilling procedure; and (**b**) experimental setup.

In order to obtain simulated force and thermal load parameters, dry drilling experiments were performed on a five-axis machining center (VMC-C30, Tuopu CNC Technology Co., Ltd., Shanghai, China) using a 6 mm diameter solid carbide drill (Sandvik-R846-0600-30-A1A, Sandvik Group, Sandviken, Sweden). The material was 10 mm thick TiBw/TC4. The drilling principle and experimental device are shown in Figure 3. The bottom of the dynamometer (KISTLER-9272, Kistler Instrumente AG, Winterthur, Switzerland) was mounted on the machine table, the upper part was connected with a three-inch vise, and the workpiece was suspended on the vise. The multi-channel signal analyzer (DH5922N, Donghua Testing Technology Co., Ltd., Shenzhen, China) was connected to a 0.5-mm diameter K-type thermocouple for measuring the real-time temperature of the workpiece. The drilling feed rate was 10 mm/min and the tool speed was 1500 r/min. The drilled hole wall was observed using a scanning electron microscope (model: JSM-6360LV).

**Figure 4:**
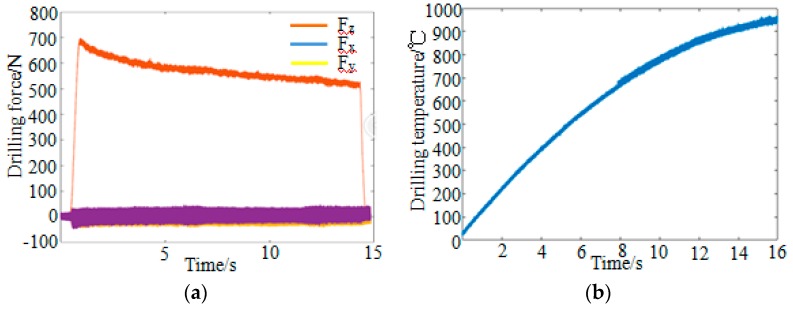
Variation of drilling force and drilling temperature. (**a**) Drilling force; and (**b**) drilling temperature.

Changes in the drilling force with time were obtained by experiment, as shown in Figure 4a. When the drilling process began, the drilling force along the *z*-axis increased rapidly until reaching a maximum value of approximately 700 N. Then, the drilling force acting in the *z*-direction slowly decreased to 500 N and remained relatively stable. The drilling forces along the *x*-axis and *y*-axis also followed this trend; the drilling force reached up to 40 N and was stable at around 25 N. The curve of workpiece temperature versus time is shown in Figure 4b. The drilling temperature measured by the analyzer linearly increased over time and the internal temperature of the material reached up to 970 °C.

### 2.3. Establishment of the Simulation Model

**Figure 5:**
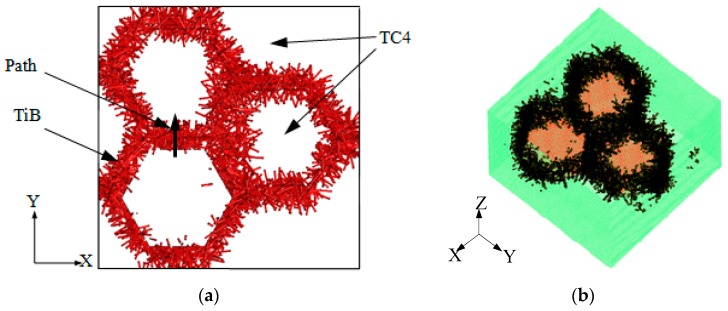
Three-dimensional model of TiBw/TC4 composite. (**a**) Representative elementary volume (RVE); and (**b**) network generation.

In view of the above conclusions, the representative volume element (RVE) of the TiBw/TC4 composite material was established by the Digimat-FE module, as shown in Figure 5a. The model was a regular hexahedron with a side length of 3 mm. White areas in the figure represent the TC4 matrix and the red needles were TiB whiskers. The whiskers were disorderly distributed and the cross section was hexagonal; its side length was 0.6 mm. The whiskers account for 8.5% of the volume of the whole model, the diameter of the whiskers was 0.045 mm, and the aspect ratio was set to 20 [19]. To guarantee precision, the mesh of the entire model was refined and the hexahedral element was chosen as the network type. The size of the network was 0.025 mm with 1,771,561 nodes and 1.728 × 10^6^ elements. Since the whiskers were located inside the material, the matrix network was hidden, as shown in Figure 5b.

To explore the changes in the microstructure of composite materials and improve simulation efficiency, the simulation of the first stage was mainly carried out. *F_z_* should linearly increase from 0 to 700 N and *F_x_* and *F_y_* linearly should increase from 0 to 40 N. The six degrees of freedom at the bottom of the model were restricted and the temperature was set to increase linearly from 25 to 150 °C.

## 3. Simulation Results Experimental Verification

### 3.1. Analysis of Microscopic Properties

**Figure 6:**
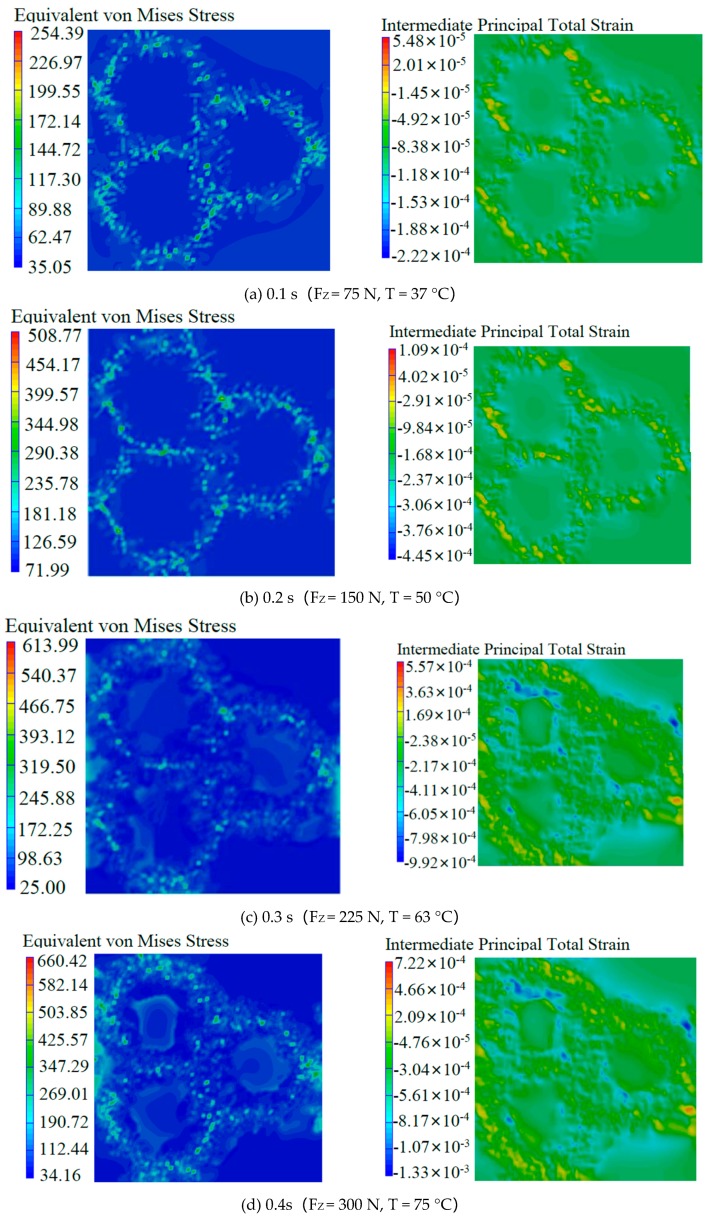

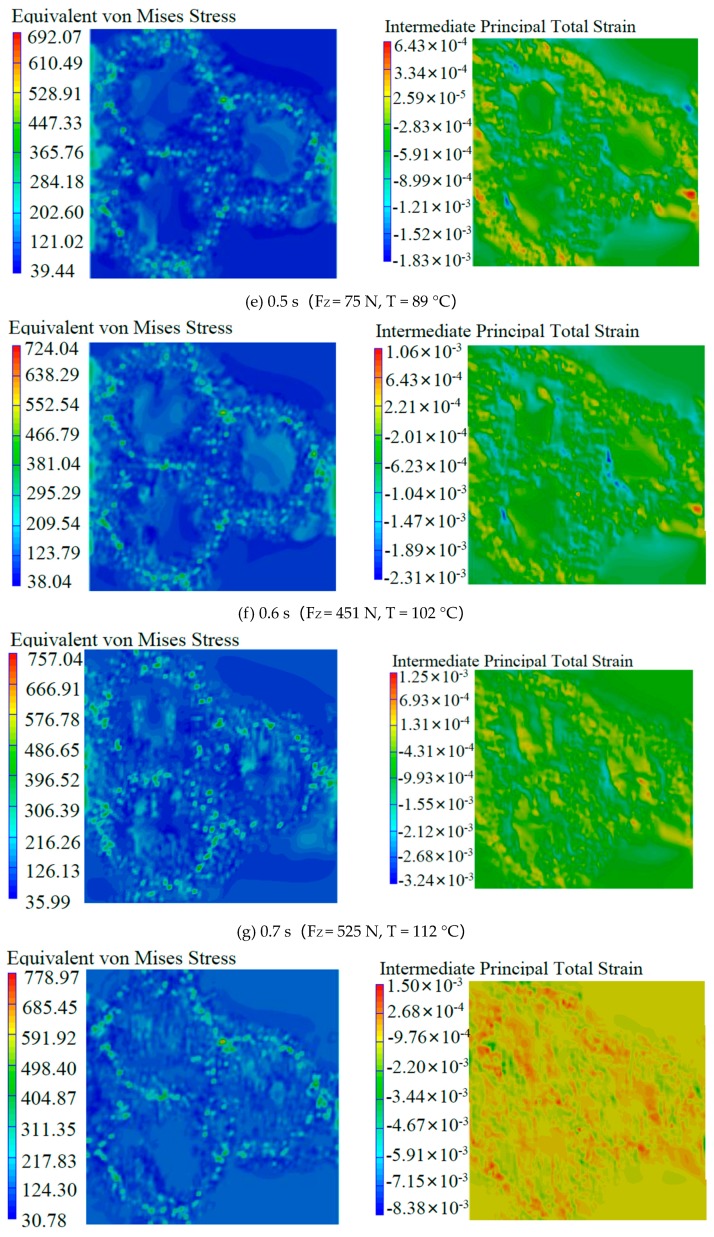

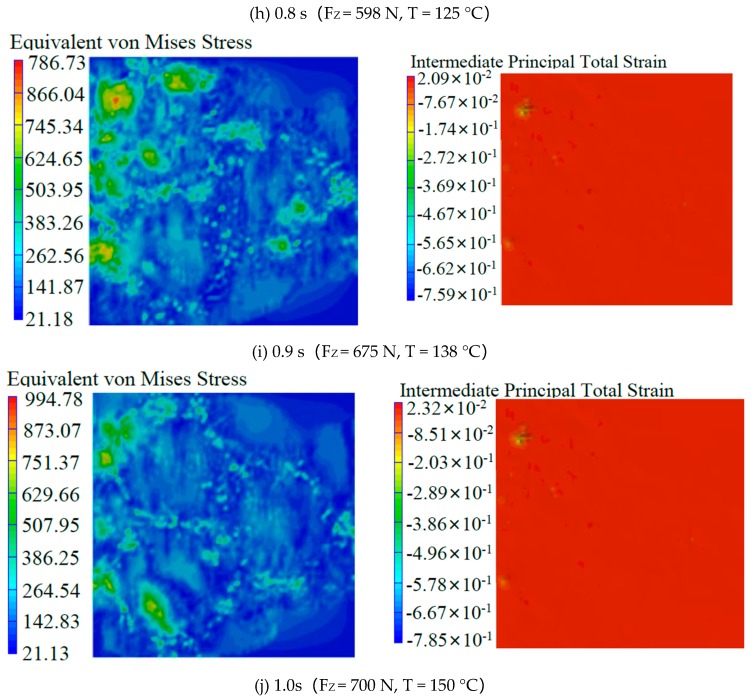
Stress and strain contour image for TiBw/TC4 composite.

The simulation results of the microscopic properties under simulated drilling loading conditions are shown in Figure 6. At the initial moment of drilling, the drilling force was about 75 N and the material was approximately 40 °C. Most of the stress was borne by the whiskers. The maximum stress reached 254 MPa; the matrix was only subjected to very small stress and the minimum stress was only 35 MPa, but the strain of the matrix was higher than that of the whiskers. The entire model was evenly stressed with no obvious changes, as seen in Figure 6a. At time 0.4, when the drilling force was about 300 N and the material was approximately 75 °C, only a small amount of stress was born by the matrix and the minimum stress was 34 MPa. The deformation occurred at the boundary of the whiskers and matrix, and strain at the junction between the whiskers and matrix reached a maximum at this moment, suggesting the junction had started to fail. The maximum strain was 0.0013. Microcracks began to form inside the material at the macro level [20], as seen in Figure 6d. At time 0.7, when the drilling force was about 525 N, the material was approximately 112 °C. The whole model started to deform and the matrix experienced almost the same amount of strain as the whiskers, indicating that the matrix had a weakened ability to protect whiskers at this moment. The internal microcracks of the material gradually diffused along the whiskers and formed large cracks at the macro level, which eventually led to the fracture of the material and the delamination in local areas [21,22], as seen in Figure 6g. At time 0.9, when the drilling force was about 675 N and the material was approximately 138 °C, the whiskers fractured and the stress concentration caused by the whiskers fracture would cause more whiskers to break, resulting in serious failure deformation of the whole material, thus the delamination of the material was intensified at the macro level [23], as seen in Figure 6i. It could be seen from the results that the maximum stress always appeared on the TiB whiskers and the minimum stress appeared in the matrix during the loading process. The material failure began at the junction of the matrix and the whiskers, then the matrix failed and, finally, the whiskers failed. Therefore, the stress during the drilling process was mainly born by the whiskers, and the matrix was more of a function of transmitting stress and protecting whiskers.

**Figure 7:**
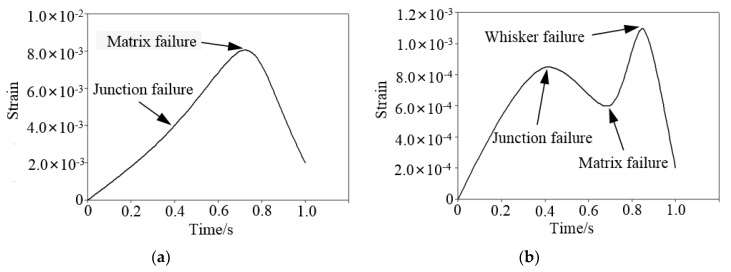
Strain curves of the composite material. (**a**) Strain in the matrix; and (**b**) strain in the whiskers.

The strain versus time curve of the material matrix and whisker is shown in Figure 7. The matrix strain is shown in Figure 7a. The junction failed at time 0.4, and the strain of the matrix rose slightly. As the drilling force was not large at this moment, it had no large impact on the overall change. At time 0.7, the matrix withstood the drilling force of more than 500 N, so it failed, and the matrix strain reached the maximum and then decreased rapidly. The whisker strain is shown in Figure 7b. There are three turning points in the curve. The first turning point appeared at time 0.4, and the junction failed at this moment. After the junction failed, the drilling force of the model was not effectively transmitted from the matrix to the whiskers. Therefore, the whiskers strain was slightly reduced. At time 0.7, the matrix could not continue to protect the whiskers after the failure, so the strain of the whiskers was greatly increased. At time 0.9, the drilling force reached 675 N, and the whiskers couldn’t withstand such a large drilling force, so the whiskers failed, and the whiskers’ strain reached the maximum, and then decreased rapidly.

### 3.2. Stress Analysis of the Junction between Whiskers and Matrix

**Figure 8:**
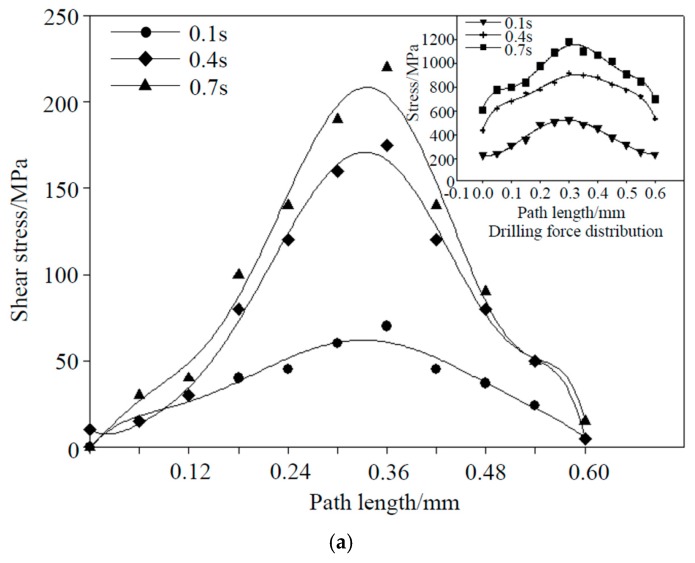

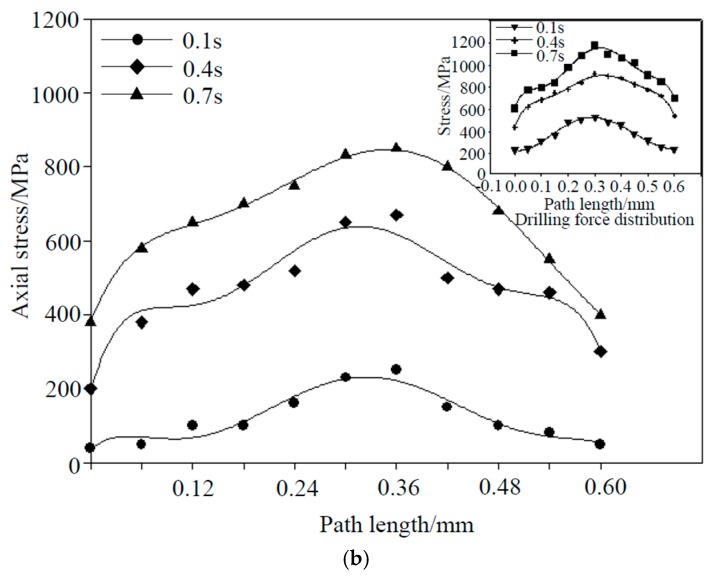
Stress distribution at the junction between whiskers and matrix. (**a**) Shear stress distribution; (**b**) axial stress distribution.

For the in situ titanium matrix composites, there is no obvious interfacial delamination between the matrix and the whiskers, so the performance of the junction is strong [17,24]. The time point and the form of junction failure will have a certain impact on the subsequent process. To ascertain the specific failure situation at the junction, we took a path in the model. The coordinates of the starting node of the path are (0.8, 1.2, 1.5), the coordinates of the end node are (0.8, 1.8, 1.5), and the total length of the path was 0.6 mm, as shown in Figure 5a. The changes in shear stress, axial stress, and total drilling force distribution at different moments on this path are shown in Figure 8.

The shear stress and total drilling force distribution are shown in Figure 8a. It can be seen from the figure that the shear stress on the whole path was symmetrically distributed, the stress on the whisker was the largest, and the force of the junction was relatively gentle, as there was no obvious stress mutation on it. At time 0.4, it can be seen that there were obvious stress mutations at both ends of the path. The stress difference at both ends of the path was inconsistent because the whiskers were disorderly distributed, and the number of whiskers and the angle of whisker insertion at both ends of the path were different. The stress distribution at both ends of the path was asymmetrical. At time 0.7, the shear stress change trend was basically the same as time 0.4, but the stress abrupt amplitude was more obvious than it, and the junction was almost completely failed.

The axial stress distribution is shown in Figure 8b. At time 0.1, the drilling force was symmetrically distributed, and the whisker bore large stress, which was basically consistent with the shear stress distribution. At time 0.4, there was a small mutation in the stress at both ends of the path. The whiskers part still bore large stress, and the whole path was roughly symmetric. At time 0.7, the stress variation of the path was intensified and the stress distribution at both ends was obviously inconsistent. This indicated that the failure conditions on both sides of the whisker were different, and the failure at the upper end of the path was more serious than the failure at the lower end of the path. From the overall situation, the distribution law of axial force distribution was basically consistent with the distribution law of total drilling force.

In summary, the whiskers part always bore large stress, the stress at the junction mainly came from the axial stress, and the proportion of shear stress was very small. However, the reason for the failure of the junction was that the shear stress of the model exceeded the connection strength between the whiskers and the matrix, and the axial force mainly acted as a squeeze and friction. The stress on the matrix could not be effectively transmitted to the whiskers after the junction failed, this led to more axial forces on the matrix. As the axial force increased, the failure process at the junction would continue to accelerate. When the junction completely failed, the matrix could not bear the axial stress alone, resulting in the compression failure of the matrix.

## 4. Experimental Verification

**Figure 9:**
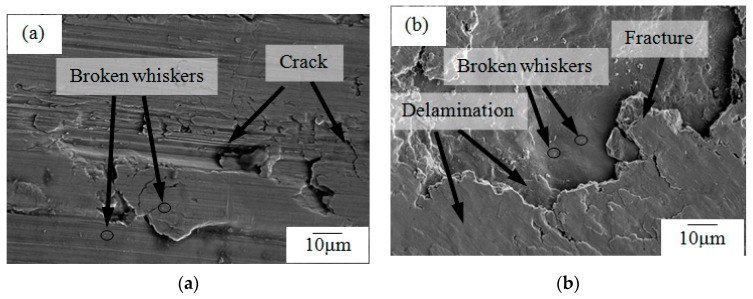
Scanning electron microscopy (SEM) images of surface morphologies of drilled hole walls of the TiBw/TC4 composite. (**a**) The entrance of the hole; (**b**) 0.15mm from the entrance of the hole.

After the drilling experiments were performed, the workpiece was dissected and the surface morphologies of the drilled hole walls of the TiBw/TC4 composite were observed. Scanning electron microscope (SEM) images of the surface morphologies are presented in Figure 9.

The surface morphology at the entrance of the hole is shown in Figure 9a, which illustrates failure modes such as cracks and a small part of the broken whiskers during the drilling process. The surface topography of about 0.15 mm from the entrance of the hole is shown in Figure 9b. It can be seen that failure modes such as cracks, delamination, and whisker breakage occur within the material structure during the drilling process. From the perspective of drilling energy, there are two ways to destroy the whisker structure during drilling: direct cutting and pulling out [25]. Whisker breakages occur when the shear stress acting in the axial direction of the whisker exceeds the ultimate shear strength of the whisker. Very small white dots on the surface of the drilled hole in the workpiece were cut whiskers. The phenomenon of pulling out was due to the shear strength exceeding the strength of the connection between the matrix and the whiskers, but the instantaneous energy was very low which was not enough to cut the whiskers. At this moment, cracks were easily formed on the hole wall of the workpiece, when the crack expanded to a certain extent, it caused the material to break. Delamination of the material is a separation and fracture phenomenon caused by external forces. Existing research indicates that the delamination of material is mainly caused by the drilling force and drilling temperature, and usually appears at the porthole of the workpiece [13]. Therefore, when the drilling force reached about 300 N, the junction between the whiskers and the matrix failed. The failure was mainly the phenomenon of pulling out, and microcracks appeared inside the material. When the drilling force reached 525 N, the TC4 matrix began to fail and large cracks and local fractures appeared inside the material. When the drilling force reached 675 N, the whiskers failed and the whiskers were cut off. In the case of whisker stress, the whiskers were more likely to be cut when the axial force was perpendicular to the direction of the whiskers. The whiskers were more likely to be pulled out when the axial force direction was parallel to the direction of the whisker, which caused large areas of material debond and peel off. Then the delamination of materials was initiated. This explains why a small part of the whiskers was broken even though it had not reached breaking strength.

The whole simulation process was based on the Tsai–Wu failure criterion. The performance degradation of the material was simulated from three aspects, namely the junction, the matrix, and the whiskers, and compared with the gradual failure process observed during practical drilling. The results of the simulation were almost consistent with the experimental results, thus validating the accuracy of the simulation model.

## 5. Study of Whisker Motion

**Figure 10:**
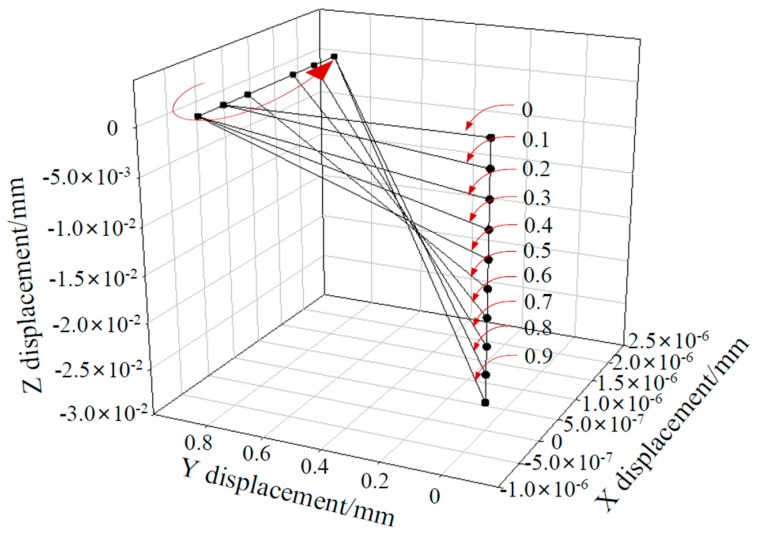
Spatial displacement of whiskers under drilling.

During the drilling process, changes in the drilling force and drilling heat will lead to very small deformations of the material, resulting in very small motions of the whiskers. Whisker motion is a direct reflection of the strain and stress in the space, and also has a subtle relationship with other phenomena, including crack formation and delamination during the drilling process [13]. A single whisker of the model was studied and the motion of the whisker displacement was determined by analyzing the node coordinates on two sides of the whisker at different moments in time, as shown in Figure 10.

As can be seen from Figure 10, one side of the whisker mainly moves along the z-direction, while another side moves in the x-direction. The side moving along the x-direction appears to be undergoing are reciprocating motion when the strain is 0.4, which induces very small angular rotations of the whisker to occur.

It can be speculated from the above analysis that failure of the interface and inconsistent stress on either side of the whisker leads to very small angular rotations of the whiskers. The side with severe failure, i.e., the side with larger stress, rotates to the other side to maintain the stability of the whisker. When the strain in the whisker rapidly increased, the displacement of the whisker was more obvious than at any other time. The strain reached a maximum when the whisker failed, and it can be seen from Figure 10 that the length of the whisker also reaches a maximum at this moment.

## 6. Conclusions

In view of the difficulty in processing titanium matrix composites and the lack of sound micromechanics research, we used Digimat multi-scale modeling technology to establish multi-scale representative voxels that could accurately reflect the structural characteristics of TiBw/TC4 composites. Taking the drilling loading as an example, the influence of the drilling force and temperature gradient on the structure of TiBw/TC4 composites was researched, and the structural failure process and failure modes of the composites were revealed.

(1) The whiskers always bore the maximum stress of the TiBw/TC4 composite during drilling, but the matrix strain was higher than the whisker strain. The junction between the matrix and the whiskers failed first (F = 300 N, T = 75 °C), then the matrix failed (F = 525 N, T = 112 °C) and, finally, the whiskers failed (F = 675 N, T = 138 °C).
(2) The junction between the matrix and the whiskers was mainly affected by the axial stress. The cause of the failure of the junction was due to the shear failure, and the axial stress accelerated the complete failure of the junction.
(3) The material structure was damaged by cracks, delamination, whiskers cutting, etc., which indicated that the FEM was consistent with experiment. It proved the accuracy and feasibility of the simulation model. Therefore, the model and the simulation method were experimentally processed. It had practical significance.
(4) During the drilling process, stress at the interface between the whiskers and matrix increases. When the stress becomes too large, the interface fails and induces whisker rotation. Subsequently, the strain in the matrix increases and the matrix fails when it reaches the peak value. At this time, the strain in the whiskers rapidly increases. After the strain in the whiskers reaches a peak value, the whiskers fail and the whiskers also reach a maximum length.

## Figures and Tables

**Table 1:** Main parameters of TiBw/TC4 composites with 8.5%volume fraction of whiskers.

Name of Parameter	TC4	TiB
Density (T/mm^3^)	4.50 × 10^−9^	4.52 × 10^−9^
Young modulus (MPa)	6.8 × 10^4^	5.65 × 10^5^
Poisson ratio	0.33	0.19
Thermal expansivity (°C^−1^)	7.89 × 10^−6^	8.10 × 10^−6^
Yield strength (MPa)	700	1100
Strength of extension *X_t_* (MPa)	855	1300
Strength of compression *X_c_* (MPa)	17.81	305
Strength of extension *Z_t_* (MPa)	860	1300
Strength of compression *Z_c_* (MPa)	967	1100
Shear strength *S* (MPa)	770	1170
Residual stiffness coefficient	0.4	0.07
